# A putative terpene cyclase gene (*CcPtc1*) is required for fungal development and virulence in *Cytospora chrysosperma*

**DOI:** 10.3389/fmicb.2023.1084828

**Published:** 2023-02-20

**Authors:** Yuchen Yang, Lu Yu, Xiaolin Qiu, Dianguang Xiong, Chengming Tian

**Affiliations:** The Key Laboratory for Silviculture and Conservation of Ministry of Education, College of Forestry, Beijing Forestry University, Beijing, China

**Keywords:** *Cytospora chrysosperma*, secondary metabolites, terpene cyclase, pathogenicity, metabonomics

## Abstract

*Cytospora chrysosperma* is a destructive plant pathogenic fungus, which causes canker disease on numerous woody plants. However, knowledge concerning the interaction between *C. chrysosperma* and its host remains limited. Secondary metabolites produced by phytopathogens often play important roles in their virulence. Terpene cyclases (TC), polyketide synthases (PKS) and non-ribosomal peptide synthetases (NRPS) are the key components for the synthesis of secondary metabolites. Here, we characterized the functions of a putative terpene type secondary metabolite biosynthetic core gene *CcPtc1* in *C. chrysosperma*, which was significantly up-regulated in the early stages of infection. Importantly, deletion of *CcPtc1* greatly reduced fungal virulence to the poplar twigs and they also showed significantly reduced fungal growth and conidiation compared with the wild-type (WT) strain. Furthermore, toxicity test of the crude extraction from each strain showed that the toxicity of crude extraction secreted by Δ*CcPtc1* were strongly compromised in comparison with the WT strain. Subsequently, the untargeted metabolomics analyses between Δ*CcPtc1* mutant and WT strain were conducted, which revealed 193 significantly different abundant metabolites (DAMs) inΔ*CcPtc1* mutant compared to the WT strain, including 90 significantly downregulated metabolites and 103 significantly up-regulated metabolites, respectively. Among them, four key metabolic pathways that reported to be important for fungal virulence were enriched, including pantothenate and coenzyme A (CoA) biosynthesis. Moreover, we also detected significant alterations in a series of terpenoids, among which (+)-ar-turmerone, pulegone, ethyl chrysanthemumate, and genipin were significantly down-regulated, while cuminaldehyde and (±)-abscisic acid were significantly up-regulated. In conclusion, our results demonstrated that *CcPtc1* acts as a virulence-related secondary metabolism factor and provides new insights into the pathogenesis of *C. chrysosperma*.

## 1. Introduction

Filamentous fungi are known to produce an abundant of bioactive secondary metabolites that involve in fungal transcription, development, intercellular communication, and virulence (Wu et al., 2012; Brakhage, 2013). Remarkably, many of secondary metabolites show a wide range of important applications in antiviral, antibiotic, antitumor, antihypercholesterolemic, and immunosuppressant activities as well as phytotoxic and mycotoxic activities (Bok and Keller, 2004; Saha et al., 2020). Phytopathogenic fungi will produce various mycotoxins during the interaction processes, which can promote the fungal colonization, induce plant cell death and cause disease (Wang et al., 2014), such as the T-toxin from *Cochliobolus heterostrophus* (Wu et al., 2012), deoxynivalenol (DON) from *Fusarium* spp. (Feizollahi and Roopesh, 2022), and aflatoxin produced by *Aspergillus flavus* (Yang et al., 2020). These secondary metabolites have been proven to be crucial for virulence and are generally synthesized by secondary metabolite biosynthetic gene clusters (SMBGCs) including the polyketide synthase type (PKS), non-ribosomal peptide synthetase type (NRPS), a dimethylallyl tryptophan synthetase type (DMATS) and terpene cyclase type (TC) (Rokas et al., 2018; Jia et al., 2019). It has been reported that the velvet family proteins (LaeA, VeA, and VelB) and cytochrome P450 related enzymes (CYP) are also contribution to the synthesis of secondary metabolites (Bok et al., 2006; Keller et al., 2006; Bayram et al., 2008).

To date, mycotoxins synthesized by SMBGCs have been implicated as crucial pathogenic factors for pathogens, such as T-toxin, Aflatoxin B_1_ (AFB_1_), cercosporin toxin, fusaoctaxin A, and botrydial (Deighton et al., 2001; Choquer et al., 2005; Siewers et al., 2005; Baker et al., 2006; Myung et al., 2012; Jia et al., 2019; Caceres et al., 2020). AFB_1_ is a toxic secondary metabolite produced by several fungal species belonging to the *Flavi* section of the *Aspergillus* genus (Caceres et al., 2016). Prevalence of the cercosporin toxin biosynthesis (*CTB*) gene clusters as virulence promoters in *Cercospora* sp. (Daub and Ehrenshaft, 2000; Choquer et al., 2005; Ebert et al., 2019). In addition, numerous studies have shown that the backbone genes of the SMBGCs (NRPS, PKS, and TC) are also strongly associate with the pathogenicity of pathogenic fungi, including *Verticillium dahliae, Pyrenophora tritici-repentis, Diaporthe helianthi, Trichoderma virens*, and *C. heterostrophus* (Baker et al., 2006; Dorrestein and Kelleher, 2006; Brakhage and Schroeckh, 2011; Crutcher et al., 2013; Ruocco et al., 2018; Rawlinson et al., 2019; Li et al., 2022). For instance, in *Alternaria alternata*, the production of HC and AM toxin, virulence factors (pathogen determinants that cause damage to the infected host), were affected when *NRPS* was deleted (Johnson et al., 2000). Moreover, PKS catalyzes the biosynthesis of polyketides, which consist of a large and diverse group of secondary metabolites, such as antibiotics, toxins, and melanin (Kim et al., 2005; Ruocco et al., 2018; Li et al., 2022). However, compared with the NRPS and PKS, there are relatively few functional studies on TC, especially in phytopathogenic fungi.

Generally, plant pathogenic fungi produce kinds of related compounds rather than a single toxin, and they are often differing in biological activity. *Fusarium graminearum*, the causal agent of *Fusarium* head blight, crown rot and seedling blight on wheat, produces several mycotoxins in the infected plants, including trichothecenes, zearalenone and fusaoctaxin A. *Valsa mali*, the pathogen of apple canker disease, can produce several toxins such as protocatechuic acid, *p-*hydroxybenzoic acid, *p-*hydroxyacetophenone, phloroglucinol, 3-(hydroxyphenyl) propionic acid, and isocoumarins, which play important roles in promoting the infection of *V. mali* on apple trees (Wang et al., 2014; Feng et al., 2020).

The necrotrophic phytopathogenic fungus *Cytospora chrysosperma* (Pers.) Fr is the causal agent of canker disease which causes serious stem damage in numerous woody plants annually, especially, leading to serious forestry, ecological and economic losses each year in northwest China (Adams et al., 2006; Kepley et al., 2015; Wang et al., 2015). Until now, several genes associated with pathogenicity were identified and functionally analyzed in *C. chrysosperma* (Wang and Wang, 2020; Han et al., 2021a,b; Wen et al., 2021). For instance, *Sge1* homolog played important roles in fungal virulence and expression of effector genes in *C. chrysosperma* (Han et al., 2021b). In addition, two conserved MAPK members in *C. chrysosperma, CcPmk1*, and *CcSlt2*, were characterized as vital pathogenicity-related regulators, which regulate fungal virulence and expression of secondary metabolism gene clusters (Yu et al., 2019, 2022a; Xiong et al., 2021). Notably, *C. chrysosperma* is considered to be a necrotrophic fungus similar to *V. mali* which tend to kill its host cells rapidly (Biggs et al., 1983; Adams et al., 2006; Yin et al., 2015). Previous studies have shown that *V. mali* synthesizes a variety of toxins to promote the infection process (Wang et al., 2014; Feng et al., 2020; Zhang et al., 2022). However, the molecular mechanism and functions of SMBGCs and backbone genes, as well as their effects on pathogenicity, is scarcely reported in *C. chrysosperma*. Lately, a total of 68 secondary metabolite core genes were predicted in *C. chrysosperma* genome including NRPS and NRPS-like, PKS, terpene, hybrid SMB, and other SMB gene models (Xiong et al., 2021). Based on these findings, we believe that revealing the function of secondary metabolic genes is important for the exploration of the molecular pathogenesis of *C. chrysosperma*.

Our previous works revealed that the whole terpene type gene cluster (*GME3317_g* to *GME3324_g*) and NRPS-T1PKS hybrid type gene clusters (*GME3437_g* to *GME3444_g*) were all significantly down-regulated in the Δ*CcPmk1* and Δ*CcSlt2*. Remarkably, the backbone gene *CcPpns1* (*GME3440_g*) of gene cluster from *GME3437_g* to *GME3444_g* was important for fungal virulence (Yu et al., 2022b). In this study, we showed that *CcPtc1*, the backbone gene of terpene type gene cluster, markedly affected morphological development, pathogenicity, and toxic secondary metabolites of *C. chrysosperma*. Moreover, the secondary metabolites secreted by *C. chrysosperma* could cause cell death in the host plants. Toxicity tests also proved that absence of *CcPtc1* caused markedly compromised toxicity of *C. chrysosperma* in comparison with wild-type (WT) strain. Furthermore, metabolomic analyses of the Δ*CcPtc1* and WT displayed large quantities of different abundant metabolites (DAMs), many of which has been reported correlated with pathogenicity in phytopathogenic fungi, such as pantothenate and trehalose 6-phosphate. In addition, deletion of *CcPtc1* also resulted in changes in terpenoids in *C. chrysosperma*. Collectively, the results increased our knowledge on the TC type secondary metabolism gene in fungal pathogenicity, which might provide new clues for the control strategies of canker disease.

## 2. Materials and methods

### 2.1. Fungal strains and cultivation conditions

The WT strain of *C. chrysosperma* (CFCC 89981) used in this study was preserved in the forest pathology laboratory of Beijing Forestry University (Fan et al., 2020). Strains used in this study were regularly cultured on potato dextrose agar medium (PDA; 20% potato extract, 2% glucose, and 1.5% agar) at 25°C. For DNA isolation and RNA isolation, mycelia were cultured in liquid potato dextrose broth medium (PDB; 20% potato extract and 2% glucose) for 2 days at 150 rpm, 25°C. *Populus* bark broth medium (PBB; 30% 1-year-old polar branch extract and 1% glucose) was used for the extraction of crude secondary metabolites.

### 2.2. Sequence and phylogenetic analysis of *CcPtc1*

The sequence of terpene type gene cluster (*GME3317_g* to *GME3324_g*) were acquired from the draft genome sequence of *C. chrysosperma*, which had been sequenced by our laboratory (NCBI GenBank accession number JAEQMF000000000). The genome sequences of the terpene type gene cluster and the corresponding CDS sequences were committed to the Gene Structure Display Server (GSDS)^1^ to analyze the number and alignment of introns and exons. TBtools (V0.66836) was used to visualize the relative expression level (Chen et al., 2020). The homologs of this terpene type gene cluster were searched in the genome of other microorganisms in the JGI database. The domain structures of terpene type gene cluster were annotated using the InterProScan tool.^2^ In addition, phylogenetic analysis was conducted with MEGA 10.0 software using the full-length protein sequences and neighbor-joining method with 1,000 bootstrap replications.

### 2.3. Targeted disruption of *CcPtc1* and mutant complementation

The split marker method was used to construct the *CcPtc1* gene deletion (Δ*CcPtc1*) mutants as previously described (Catlett et al., 2003; Goswami, 2012). According to this method, the upstream (∼1.3 kb) and downstream (∼1.2 kb) flanking sequences of *CcPtc1* were amplified by primer pairs CcPtc1-5Ffor/CcPtc1-5Frev and CcPtc1-3Ffor/CcPtc1-3Frev, respectively (Supplementary Table 1). The hygromycin B resistance cassette (HPH) was amplified by specific primer pairs hygromycinfor and hygromycinrev, which included approximately 20 bp overlapped 5′ and 3′flanking sequences, respectively. Then, the resulting upstream and downstream fragments were fused with two-thirds of the hygromycin B resistance cassette by overlap PCR using primer pairs CcPtc1-5Ffor/HY-R and YG-F/CcPtc1-3Frev, respectively. The two overlapping fragments were directly transformed into protoplasts of the WT strain by using the PEG-mediated transformation, and the transformants were selected on TB_3_ agar medium supplemented with 20 μg/ml hygromycin B. All transformants were identified by PCR assays with the primer pairs External-CcPtc1for/External-CcPtc1rev and Internal-CcPtc1for/Internal-CcPtc1rev to screening the successful replacement transformants. In addition, to analyze homologous recombination events in the transformants, southern blotting analysis was performed with the DIG High Prime DNA Labeling and Detection Starter Kit I, following the manufacturer’s protocol (Roche, Germany). *BglI* was used to digest the genomic DNA extracted from the WT strain and the transformants. The probes were amplified by the primers ProbeHPHfor and ProbeHPHrev from *HPH* and the primers ProbeCcPtc1for and ProbeCcPtc1rev for *CcPtc1*.

To generation the *CcPtc1* gene complementation construct, a fragment containing the entire length of the *CcPtc1* coding region along with native promoter sequence and terminator sequence was cloned from gDNA using the primer pair CcPtc1-Compfor/CcPtc1-Comprev. The resulting PCR products were co-transformed into protoplasts of the Δ*CcPtc1*-11 strains with a geneticin-resistant cassette. After that, we selected the transformants in TB_3_ medium supplemented with 40 μg/ml geneticin. Successful complementation was confirmed by PCR with the primer pair Internal-CcPtc1for/Internal-CcPtc1rev. The complementation strain was named Δ*CcPtc1/PTC1* in this study. All primers used in gene deletion and complementation were listed in Supplementary Table 1.

### 2.4. Fungal growth and conidiation

To analyze the differences in vegetative growth and conidiation among the WT, deletion mutants, and complemented strains, each strain was inoculated on the PDA plates at 25°C in the dark. The growth and conidial formation were observed at 24, 48, and 60 h and 30 days post inoculation. Each experiment was replicated at least three times.

### 2.5. Pathogenicity assays

Healthy annual poplar twigs collected from the nursery garden in the Beijing forestry university were used for pathogenicity assay. Hyphal plugs of the WT, gene deletion mutants, and complemented strains were inoculated on the 20-cm-long twigs which scorched with a flat iron (5 mm in diameter). The inoculated twigs were incubated at 25°C for 5∼8 days under moist conditions and photographs were taken after 5 days. The experiments were repeated at least three times.

### 2.6. Extraction of crude extracts and toxicity tests

For analysis of metabolite production, the plugs of WT and Δ*CcPtc1* were cultivated into PBB medium at 25°C and 150 rpm for 10 days. Culture filtrates of WT, Δ*CcPtc1*, and PBB medium were extracted with equal volume of ethyl acetate for three times, and the organic phase was saved. Organic phases were combined to obtain crude extracts of secondary metabolites. The organic phases were distilled using a rotary evaporator under 45°C and re-dissolved in 20 ml Dimethyl sulfoxide (DMSO). The phytotoxic activities of the crude extracts were tested on polar leaves using the leaf puncture method. PBB crude extract–treated and DMSO-treated samples were kept as control. Inoculated leaves were incubated in a petri dish under 25°C. The pictures were taken at 24 h after treatment. The experiment was performed with three biological replicates at least.

### 2.7. RNA extraction and quantitative RT-PCR

To analyze the expression levels of other genes among the terpene type gene cluster in the *CcPtc1* deletion mutant, the WT, Δ*CcPtc1-*11 and Δ*CcPtc1-*14 strains were cultivated in PDB supplemented with sterilized poplar twigs at 25°C and 150 rpm for 48 h. Mycelium was harvested by Miracloth (Calbiochem). Then flash-frozen in liquid nitrogen and ground to powder. Total RNA was extracted from powder using RNA Easy Fast Plant Tissue Kit (TIANGEN, China) according to the manufacturer’s protocol. First-strand cDNA was prepared using ABScript II cDNA Fist-Strand Synthesis Kit (ABclonal, China).

Quantitative real-time PCR was performed with SuperReal PreMix Plus (ABclonal, China) on the Applied Biosystems 7500 Real-Time PCR system (Applied Biosystems). The *CcActin* gene of *C. chrysosperma* was used as an internal reference for all RT-qPCR experiments. Relative expression was calculated using the 2^–Δ Δ^ *^Ct^* method. The experiment was performed in triplicate with three independent technical replicates each. All primers used in the present study were listed in Supplementary Table 1.

### 2.8. Analysis of metabolomics data

To prepare the metabolome samples, the WT and Δ*CcPtc1* strains was simultaneously cultured in PBB medium with the same conditions as crude extraction assay. Each strain contained six repeats and generated twelve datasets.

All samples were placed in the EP tubes and resuspended with prechilled 80% methanol by well vortex. After whirled (melted on ice for 30 s), sonification (6 min), and centrifuged (5,000 rpm, 4°C for 1 min), the supernatant of all samples was freeze-dried and dissolved with 10% methanol. Finally, the solution was injected into the LC-MS/MS system for further analysis. UHPLC-MS/MS was used to analyze the composition of the WT and Δ*CcPtc1* strains using a Vanquish UHPLC system (Thermo Fisher, Germany) coupled with an Orbitrap Q ExactiveTM HF mass spectrometer (Thermo Fisher, Germany) in Novogene Co., Ltd. (Beijing, China). Samples were injected onto a Hypesil Gold column (100 × 2.1 mm, 1.9 μm) with a 12-min linear gradient at a flow rate of 0.2 mL/min. Q ExactiveTM HF mass spectrometer was operated in positive/negative polarity mode with spray voltage of 3.5 kV, capillary temperature of 320°C, sheath gas flow rate of 35 psi and aux gas flow rate of 10 L/min, S-lens RF level of 60, Aux gas heater temperature of 350°C.

The raw data generate by UHPLC-MS/MS were processed using the Compound Discoverer 3.1 (CD3.1, Thermo Fisher) to perform peak alignment, peak picking, and quantitation for each metabolite. The KEGG database,^3^ Human Metabolome Database (HMDB) database,^4^ and LIPIDMaps database^5^ were used to annotate these metabolites. Principal components analysis (PCA) and Partial least squares discriminant analysis (PLS-DA) were performed at metaX. We applied univariate analysis (*t*-test) to calculate the statistical significance (*p*-value). Metabolites with Variable Importance in Projection (VIP) > 1, fold change (FC) > 1.2 or FC < 0.833, and *p*-value < 0.05 were considered to be DAMs. The functions of these metabolites and metabolic pathways were annotated using the KEGG database. Enrichment metabolic pathways of differential metabolites were selected with *x*/*n* > *y*/*N* and *p* < 0.05.

## 3. Results

### 3.1. Identification of terpene type gene cluster in *C. chrysosperma*

**FIGURE 1:**
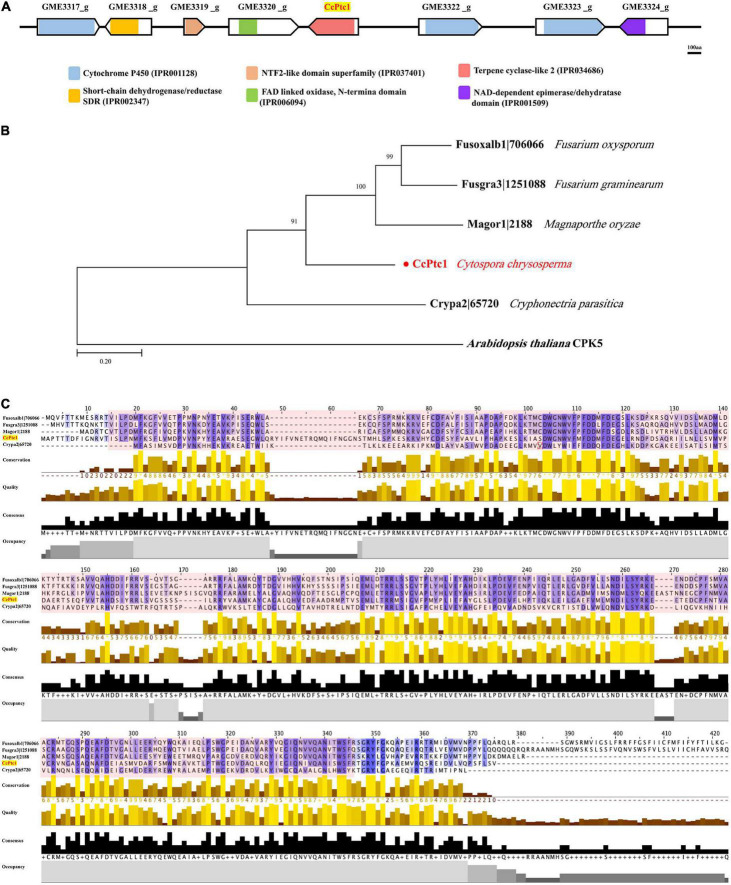
Domain and phylogenetic analysis of terpene type gene cluster (*GME3317_g* ∼ *GME3324_g*). **(A)** The line with the arrow indicates the scheme of the terpene type gene cluster (*GME3317_g* ∼ *GME3324_g*) proteins; domains were predicted using the InterProScan tool. Different color boxes indicate different domain categories. Bar = 100 aa. **(B)** Phylogenetic analysis of the backbone gene *CcPtc1* homologs from four fungi. Bootstrap percentages over 50% are indicated at the nodes. The tree was rooted in outgroup taxon *Arabidopsis thaliana*. The detailed information of the orthologs is listed in Supplementary Table 2. **(C)** Multiple sequence alignment of terpene cyclase-like 2 protein from four fungi species. The protein domain (IPR 034686) is shadowed in red. The detailed information of the terpene cyclase-like 2 protein orthologs are listed in Supplementary Table 2.

Our previous works found that two putative secondary metabolism related gene clusters (*GME3437_g* to *GME3444_g* and *GME3317_g* to *GME3324_g*) were regulated by the key pathogenicity factor *CcPmk1* and *CcSlt2*. In this study, the backbone gene (*GME3321_g*) of gene cluster from *GME3317_g* to *GME3324_g* was functional characterized. *GME3321_g* contained a terpene cyclase-like 2 protein family domain (IPR 034686), therefore, we named it as *CcPtc1* (Figure 1A). Additionally, *GME3317_g, GME3322_g*, and *GME3323_g* contains a conserved cytochrome P450 domain (IPR 001128), respectively (Figure 1A). The structure of the 8 genes among these terpene type gene cluster were characterized using the GSDS, which contained 0 to 7 introns (Supplementary Figure 1). In addition, we aimed to determine the putative homologs of this gene cluster in other fungal species. Therefore, the whole sequence of this gene cluster was used as queries to search through the genome sequences of *Magnaporthe oryzae, V. dahliae, Botrytis cinerea, Cryphonectria parasitica, Saccharomyces cerevisiae, Sclerotinia sclerotiorum*, and *Fusarium* species. Nevertheless, no significant hits were obtained in these fungal species.

Subsequently, we only identified the homologs of backbone gene *CcPtc1* in other fungal species, and many homologs were found because of the conserved sequence of terpene cyclase-like 2 protein family domain. Homologos protein sequences of *CcPtc1* were downloaded from four fungal species, including *M. oryzae, Fusarium oxysporum, F. graminearum*, and *C. parasitica*, to produce a phylogenetic tree (Figure 1B). The information of all these proteins were listed in Supplementary Table 2. Moreover, we analyzed the sequences and protein domain (IPR 034686) of terpene cyclase-like 2 proteins in these four fungi (Figure 1C). The overall amino acid identity of CcPtc1 to the Magor1| 2188, Fusoxalb1| 706066, and Fusgra3| 1251088 is with an identity of 44, 48, and 45%, respectively (Supplementary Table 2).

### 3.2. The expression of terpene type gene cluster is induced during the early infection process

**FIGURE 2:**
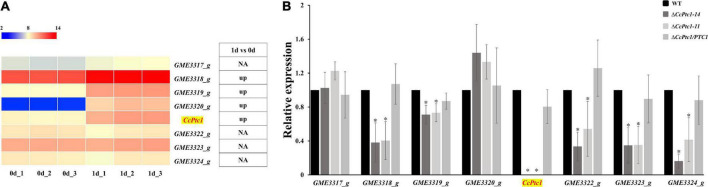
The expression patterns of terpene type gene cluster at the initial infection stages. **(A)** The heatmap shows the expression data of the genes in the cluster at the initial stages of infection. The original fragments per kilobase per million (FPKM) values of the cluster genes were transformed by log2. The color scale ranging from blue to red indicates increasing expression levels. The differentially expressed genes (|log2foldchange| ≥ 1, *p*-value < 0.05) are indicated. The “up” represents significantly up regulated. **(B)** Expression of terpene type gene cluster in wild-type (WT) and *CcPtc1* deficient mutants. RT-PCR was used to determine the cluster genes expression levels in the WT and two Δ*CcPtc1* deletion mutant strains on PDB (supplemented with sterilized poplar twigs to mimic the states of infection) at 2 dpi. The *CcActin* gene was used as the reference gene. The error bars represent the standard error based on three independent biological replicates with three technical replicates each. The data were analyzed using one way ANOVA and Duncan’s range test. The asterisks indicate significant differences (*p*-value < 0.05).

To investigate the expression of terpene type gene cluster during infection processes, we collected the expression data for these cluster genes from our previous transcriptome data of the initial infection process [0 and 1 days post-inoculation (dpi)] in poplar branches (Li et al., 2021). The expression levels of genes in this terpene type gene cluster were shown in Figure 2A, and half of the genes (4/8) were significantly upregulated at 1 dpi compared to 0 dpi. We then analyzed the expression levels of these cluster genes during the middle and late *C. chrysosperma* infection process (7 dpi and 15 dpi) of poplar branches. As shown in Supplementary Figure 2, most of the genes (7/8) were significantly upregulated at 7 dpi compared to 0 dpi, and 3 out of 8 genes were significantly upregulated at 15 dpi compared to 0 dpi. Notably, the expression of the backbone gene (*CcPtc1*) was significantly up-regulated during the whole infection processes.

In order to reveal the functions of *CcPtc1* during the infection processes, we generated two *CcPtc1* deletion mutants Δ*CcPtc1-11* and Δ*CcPtc1-14*, which had been confirmed by the PCR and Southern blot analysis (Supplementary Figures 3A, B). For the complementation of Δ*CcPtc1*, a fragment containing the native promoter and the *CcPtc1* coding sequence was isolated from genomic DNA and transformed into the Δ*CcPtc1-11* strain and screened by PCR (Supplementary Figure 3C). One complemented strain (Δ*CcPtc1*/*PTC1*) and two mutant strains (Δ*CcPtc1-11* and Δ*CcPtc1-14*) were used for subsequent phenotypic analyses.

To determine the effects of *CcPtc1* on the other genes among its cluster (*GME3317_g* to *GME3324_g*), we analyzed the expression levels of these genes in the WT and Δ*CcPtc1* strains (Figure 2B) in mimetic infection process. The qRT-PCR assays show that, 5 out of 7 genes in this cluster exhibited significantly reduced expression levels in the *CcPtc1* deletion mutants compared with those in the WT strain. These results suggest that this terpene type gene cluster, especially the backbone genes *CcPtc1*, may play important roles in the fungal pathogenicity of *C. chrysosperma*.

### 3.3. *CcPtc1* is indispensable for hyphal growth and conidiation

**FIGURE 3:**
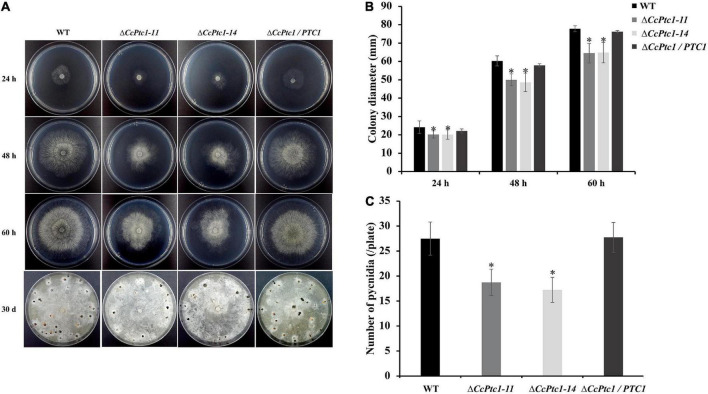
Growth and conidiation phenotype of *Cytospora chrysosperma* wild-type (WT) and Δ*CcPtc1*. **(A)** Colony morphologies and pycnidia formation of the WT, mutant, and complemented strains after 24, 48, and 60 h and 30 days of growth on PDA plates. **(B)** Bar chart showing the colony diameter strains on PDA plates. **(C)** Quantification of pycnidia production in four strains based on three independent experiments. In bar graphs, error bars represent the standard error based on three independent biological replicates with three technical replicates each. The data were analyzed using Duncan’s range test. The asterisks indicate significant differences (*p*-value < 0.05). All pathogenicity experiments were performed three times.

To determine the potential roles of *CcPtc1* in *C. chrysosperma* vegetative growth, we cultivated the WT, *CcPtc1* deletion mutants and complemented strains on PDA plates at 25°C for 60 h. Development phenotypic analysis showed that Δ*CcPtc1* exhibited an apparent smaller colony diameter compared with the WT, and the growth defects of the knockout strains were restored in complementation strain (Figures 3A, B). Compared with the WT and Δ*CcPtc1*/*PTC1* strains, the *CcPtc1* deletion mutant showed approximately 17.13% reduction in hyphal growth on PDA plates.

To investigate whether *CcPtc1* contributed to conidiation, we then calculated the number of pycnidium produced by the WT strain and *CcPtc1* deletion mutants. The results suggest that Δ*CcPtc1* produced significantly decreased amount of pycnidia compared to WT strain on PDA after 30 days (a reduction of approximately 34.5%) (Figures 3A, C). Taken together, these results suggest that *CcPtc1* is required for conidiation and fungal growth.

### 3.4. *CcPtc1* is required for fungal virulence

**FIGURE 4:**
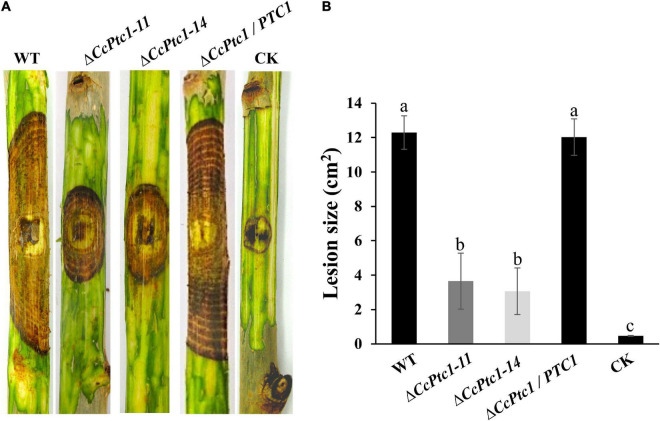
Infection phenotype of *Cytospora chrysosperma* wild-type (WT) and Δ*CcPtc1*. **(A)** Pathogenicity test of Δ*CcPtc1* on detached poplar twigs. WT, *CcPtc1* deletion strain, complemented strain Δ*CcPtc1*/*PTC1* and CK (PDA plugs) were inoculated onto detached twigs of poplar and incubated at 25°C. Typical twigs were photographed at 7 dpi. **(B)** The bar chart shows average lesion size of twigs quantified at 7 dpi and means ± SD. CK means inoculating PDA plugs after scalding. The error bars represent the standard errors based on three independent biological replicates with three technical replicates each. The data were analyzed using Duncan’s range test. The different letters indicate significant differences from the WT strain (*p*-value < 0.05).

As mentioned above, the expression level of *CcPtc1* was significantly increased during the early infection stages. Thus, it prompted us to explore the roles of *CcPtc1* in fungal virulence. We performed a pathogenicity test on detached poplar twigs with inoculating mycelial plugs of the WT, *CcPtc1* deletion mutants, and complemented strains. As shown in Figure 4A, poplar twigs inoculated with WT strains exhibited severely typical symptoms at 4∼7 dpi, while the poplar twigs inoculated with Δ*CcPtc1* deletion mutants showed significantly reduced lesion areas (over 70% reduction). In addition, the complemented strain Δ*CcPtc1*/*PTC1* displayed a comparable lesion size as the WT strain (Figures 4A, B). Overall, these results demonstrate that the *CcPtc1* contributes to fungal virulence during the infection process.

### 3.5. *CcPtc1* affects the production of toxic secondary metabolites in *C. chrysosperma*

*CcPtc1* was predicted as the backbone gene of the secondary metabolite gene cluster and it was required for fungal virulence, thus we speculated whether the impaired pathogenicity may result from the reduced production of secondary metabolites. To investigate the role of *CcPtc1* in the production of secondary metabolites, toxicity tests were conducted. We collected culture filtrates of WT strain, Δ*CcPtc1* strain, and uninoculated medium to obtain crude extracts of secondary metabolites. Toxicity of these crude secondary metabolites was tested on poplar leaves.

**FIGURE 5:**
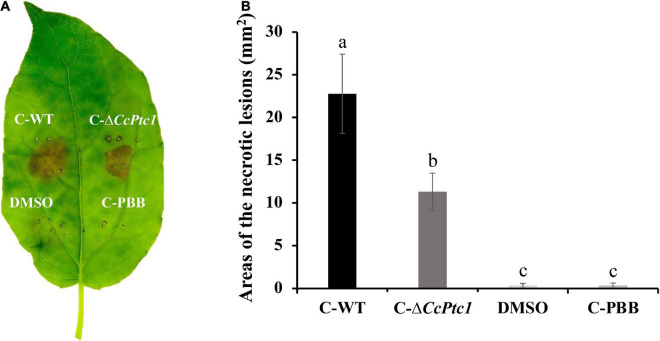
Detection of crude secondary metabolites produced by wild-type (WT; *Cytospora chrysosperma*) and Δ*CcPtc1* in infected poplar tissues. **(A)** Representative images and measurements of lesions on poplar leaves infected by indicated crude secondary metabolites at 24 h. C-WT, crude secondary metabolites of WT; C-PBB, crude extracts of PBB medium; C-Δ*CcPtc1*, crude secondary metabolites of Δ*CcPtc1*. DMSO and crude extracts of PBB were used as controls. **(B)** Bar graphs show the statistical analysis of at least three biological replicates, means ± SD are shown. The different letters indicates a significant difference (*p*-value < 0.05) based on one-way ANOVA followed by Duncan’s range test.

Infiltration the crude extracts of WT and Δ*CcPtc1* into poplar leaves with leaf puncture method produced a brown ovoid region of necrosis which extend over the entire infiltration zone at 24 h after treatment (Figure 5A). On the contrary, the DMSO solvent and uninoculated medium control did not produce any necrotic symptom. Although necrotic symptoms were similar across WT and *CcPtc1* deletion mutants, crude secondary metabolites from WT were more effective than those from Δ*CcPtc1*, the area of the necrotic lesions inoculated with the Δ*CcPtc1* was reduced by approximately 50.3% compared with the WT strain (Figures 5A, B).

These findings verify that *C. chrysosperma* was likely to produce toxic secondary metabolites which cause damage on host plant tissue, and deletion of *CcPtc1* may lead to reduction toxicity of secondary metabolites.

### 3.6. Metabolomics analysis between the wild type and the *CcPtc1* deletion mutant

**FIGURE 6:**
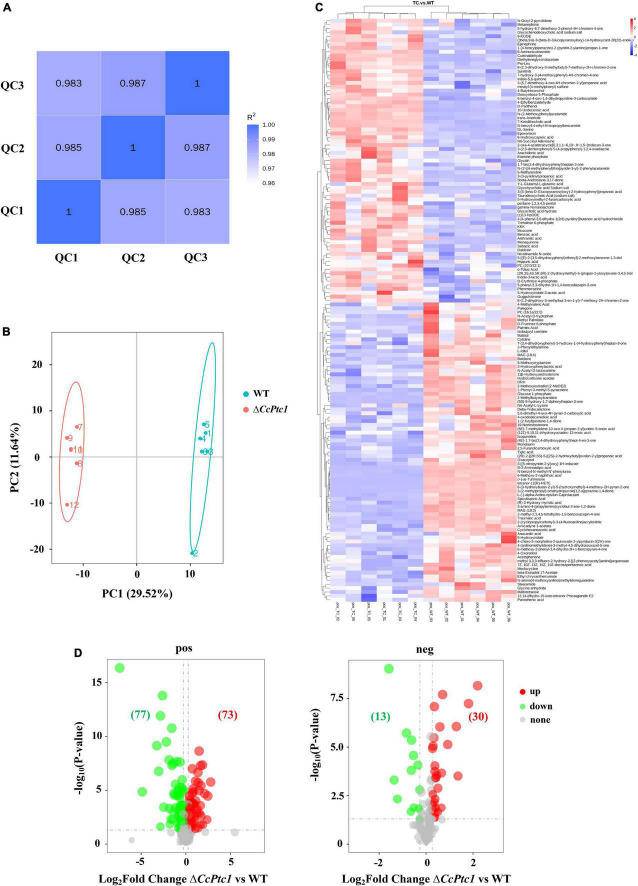
Quantity analyses of metabolomic data between wild-type (WT) group and Δ*CcPtc1* group. **(A)** In positive polarity mode, pearson correlation of three quality control (QC) samples during the LC-MS/MS analysis. **(B)** In positive polarity mode, score plot (PC1 vs. PC2) of principal component analysis (PCA) of metabolites extracted from Δ*CcPtc1* compared to those from WT strain. The green circle indicates WT group, whereas the red circle indicates Δ*CcPtc1* group. **(C)** Heat map of detected metabolites in positive polarity mode. Red and blue colors indicate increase and decrease of metabolite levels relative to the median metabolite level, respectively. **(D)** The volcano map of metabolites profile in Δ*CcPtc1* compared to those in WT strain. The increased metabolites were marked in red dots, whereas the decreased metabolites were marked in green dots. The significantly changed metabolites were defined as Variable Importance in Projection (VIP) > 1, FC (fold change) > 1.2 or *p*-value < 0.05, and FC < 0.833.

To clarify the differences in metabolites after the deletion of *CcPtc1*, six biological individuals for WT or Δ*CcPtc1* strain were analyzed by mass spectrometry-based metabolomics, respectively. High Pearson correlation values between the quality control samples were obtained, indicating high data stability during LC-MS/MS analysis (Figure 6A). The markedly separation between WT group and Δ*CcPtc1* group was validated by PCA with the first principal component (PC1) value of 29.52% and the second principal component (PC2) accounted for 11.64% of the variation (Figure 6B). The PCA analysis revealed that the metabolites of Δ*CcPtc1* and WT strains were significantly different.

**FIGURE 7:**
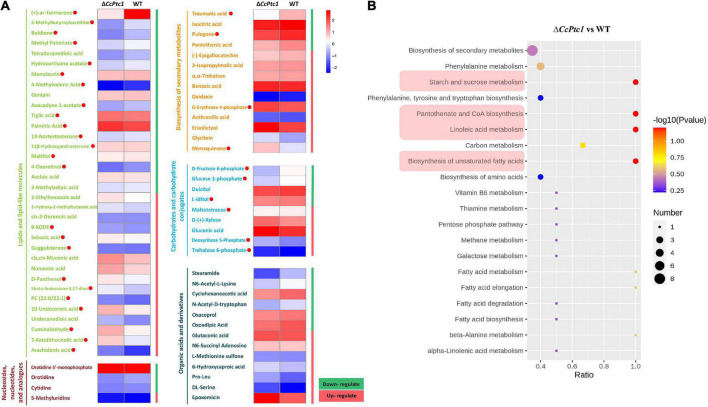
Metabolites enrichment data among wild-type (WT) and Δ*CcPtc1*. **(A)** Differential metabolites can be divided into five categories: Lipids and lipid-like molecules, Nucleosides, nucleotides, and analogs, Biosynthesis of secondary metabolites, Carbohydrates and carbohydrate conjugates and Organic acids and derivatives. Red dots represent lipid (27 metabolites, 3 was included in biosynthesis of secondary metabolites) and carbohydrates metabolites (7 metabolites, 1 was included in biosynthesis of secondary metabolites) that were significantly enriched in the positive mode. **(B)** Metabolic pathway enrichment analyses. Four metabolic pathways with lowest *p*-value were shadowed in red. The scatterplot showed the enriched KEGG pathway of differentially changed metabolites.

Here, 725 metabolites (data have been de-duplicated) were detected with varied abundance in both strains including 550 metabolites in positive polarity mode (Figure 6C) and 175 metabolites in negative polarity mode (data not shown). Among them, the abundance of 193 metabolites were significantly changed including 90 metabolites significantly decreased their abundance and 103 metabolites significantly increased their abundance (Figure 6D). According to KEGG, HMDB, or LIPID MAPS databases, these 193 DAMs were categorized to the following major classes: biosynthesis of secondary metabolites (14 annotations), nucleosides, nucleotides, and analogs (4 annotations), organic acids and derivatives (14 annotations, 1 annotation was included in biosynthesis of secondary metabolites), carbohydrates and carbohydrate conjugates (10 annotations, 1 annotation was included in biosynthesis of secondary metabolites), and lipids and lipid-like molecules (38 annotations, 4 annotations were included in biosynthesis of secondary metabolites) (Figure 7A and Supplementary Table 3). Importantly, lipid and organic acids were the category that enriched by most of DAMs in response to *CcPtc1* deletion. These observations suggest that different abundant metabolites are resulted from *CcPtc1* deletion and might be the agents for *CcPtc1* to modulate fungal phenotypes.

### 3.7. Variations in metabolic profiles in the absence of *CcPtc1*

Functionally annotation of the 193 DAMs revealed that four metabolic pathways of the *C. chrysosperma* were identified including starch and sucrose metabolism, linoleic acid metabolism, pantothenate and coenzyme A (CoA) biosynthesis, and biosynthesis of unsaturated fatty acids (Figure 7B).

**FIGURE 8:**
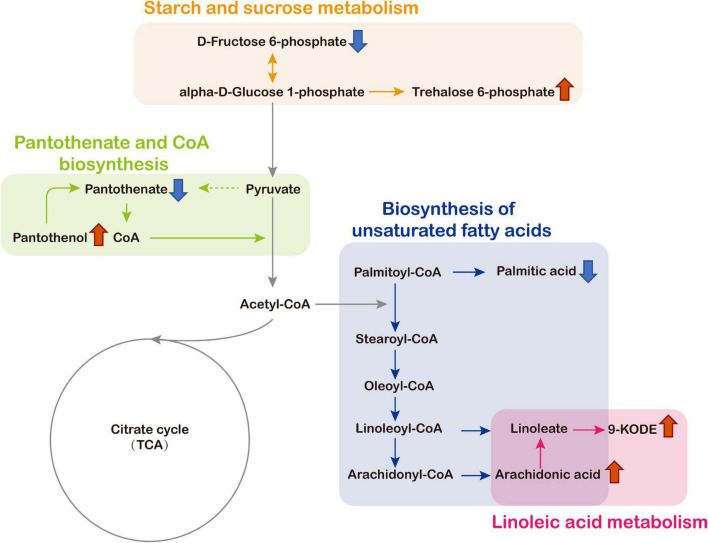
Alterations of metabolite levels in *Cytospora chrysosperma* induced by deletion of *CcPtc1* and schematic biosynthetic pathways map of these metabolites according to the KEGG database. Red and blue arrows denote, respectively, higher and lower levels of metabolites in *C. chrysosperma*. TCA, tricarboxylic acid.

The schematic map of significant enrichment metabolic pathways and changes level of key metabolites identified in the mycelia culture filtrates was shown in Figure 8. The key metabolites included trehalose 6-phosphate, D-Fructose 6-phosphate, arachidonic acid, 9-KODE, palmitic acid, pantothenate, and pantothenol were significantly influenced by *CcPtc1* (Figure 8). As shown in Figure 8, deletion of *CcPtc1* resulted in a decrease in the content of D-Fructose 6-phosphate, pantothenate and palmitic acid, and an increase in the content of trehalose 6-phosphate, 9-KODE, arachidonic acid, and pantothenol.

**FIGURE 9:**
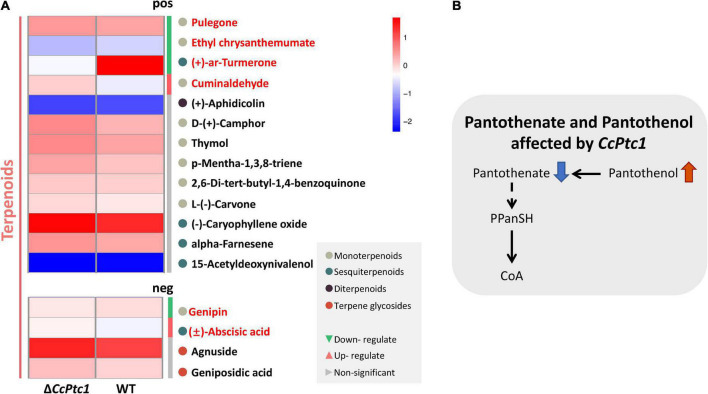
Metabolomic analysis. **(A)** The heatmap showing the full range of detected terpenoids. The red font indicates significantly enriched terpenoids. **(B)** A proposed model for the pantothenate and coenzyme A (CoA) mechanism of *Cytospora chrysosperma*. PPanSH:4′-Phosphopantetheine. The red upward arrows indicate upregulation, and the blue downward arrows indicate downregulation.

Moreover, to explore the changes of terpenoids in Δ*CcPtc1* mutants, we further analyzed the metabolomic data and identified 17 terpenoid related metabolites (Supplementary Table 4), which could be categorized into four major categories: diterpenoids, monoterpenoids, sesquiterpenoids, and terpene glycosides. Among the 17 terpene metabolites detected, the number of monoterpenoids and sesquiterpenoids was the largest, of which 9 metabolites belonged to monoterpenoids and 5 metabolites belonged to sesquiterpenoids (Figure 9A and Supplementary Table 4). Further analysis revealed that 6 terpenoid related metabolites were significantly alterations in the Δ*CcPtc1* compared to the WT, including down-regulated metabolites, (+)-ar-turmerone, pulegone, ethyl chrysanthemumate and genipin, and up-regulated metabolites cuminaldehyde and (±)-abscisic acid (Figure 9A).

### 3.8. Pantothenate biosynthesis regulated by *CcPtc1* in *C. chrysosperma*

We focused on the biosynthesis of pantothenate and CoA which are significantly enriched in positive polarity mode. Remarkably, we discovered that early intermediates of the pantothenate and CoA biosynthesis pathway (pantothenate and pantothenol) were significant changed in Δ*CcPtc1*: the amounts of pantothenate was significant down-regulated, and in contrast, the amounts of pantothenol was remarkably up-regulated compared to the WT strain. Furthermore, the Δ*CcPtc1* strain showed significant perturbations of pantothenate. Previous studies have showed that pantothenate plays dominant role in regulation of carbohydrate, lipid, and nucleic acid metabolism. As shown in Figure 6 and Supplementary Table 3, the lipid metabolites accounted for the largest number (27 out of 79) (Figure 6 and Supplementary Table 3). Besides, a certain amount of carbohydrates metabolites was also enriched (7/79) (Figure 6 and Supplementary Table 3). Importantly, the lipid (16/27) and carbohydrate (4/7) metabolites were generally reduced in *CcPtc1* mutant. It is suggested that deletion of *CcPtc1* could reduce the accumulation of pantothenate, which resulted in a significant changed in the content of lipid and carbohydrates metabolites (Figure 9B).

In this study, we found that *CcPtc1* markedly affected fungal development, pathogenicity, and toxic secondary metabolites in *C. chrysosperma*. Taken together, these results shows that *CcPtc1* acts as a crucial virulence factor.

## 4. Discussion

Plant pathogenic fungi are usually capable of secreting multifarious secondary metabolites (also known as natural products), including mycotoxins and secretory proteinaceous toxins, which subvert plant defense responses and contribute to the pathogenicity on plant hosts (Howlett, 2006). We characterized the main functions of the backbone gene *CcPtc1* of a terpene type secondary metabolism gene cluster (*GME3317_g* to *GME3324_g*), which was required for fungal growth, conidial development, biosynthesis of secondary metabolites in *C. chrysosperma*. We further performed untargeted metabolomic analysis of the WT and Δ*CcPtc1*, which revealed that *CcPtc1* was involved in the starch and sucrose metabolism, pantothenate and CoA biosynthesis, linoleic acid metabolism and biosynthesis of unsaturated fatty acids. Collectively, the results suggest that *CcPtc1* is an important pathogenic factor of *C. chrysosperma*.

Secondary metabolites secreted by some fungal pathogens often play an important role in triggering these responses (Howlett, 2006). Among them, terpenoids account for a large proportion of secondary metabolites, and the reactions catalyzed by TC are largely responsible for the chemical diversity of terpene natural products (Dickschat, 2016). In this study, we explored the function of a putative terpene cyclase, *CcPtc1*, and confirmed its ability to regulate pathogenicity, fungal development, and toxic secondary metabolites in *C. chrysosperma*. Although there are few reports on TC, this gene still plays an important role in fungal biological processes. For example, *Fusarium* mycotoxin biosynthetic genes discovered to date included a TC gene cluster for trichothecenes (Desjardins and Proctor, 2007). Moreover, in the biocontrol fungus *T. virens*, the experimental evidence demonstrates that putative terpene cyclase *vir4* and *vir* cluster was involved in the synthesis of volatile terpene compounds (Crutcher et al., 2013). As a consequence, we believe that the study of TC function is a promising direction not only in the study of pathogenic mechanism in *C. chrysosperma*, but also for filamentous plant fungi. Future investigation of the specific functions of the genes in this cluster will help us understand the assembly line of secondary metabolites synthesized by this cluster and further elucidate the molecular strategies used by *C. chrysosperma* for successful invasion.

Previous research found secondary metabolic pathways were usually capable of regulating the fungal developmental program not only including hyphae development, but also sexual fruiting-body formation (Bayram et al., 2008; Jiang et al., 2011). Meanwhile, a relationship between mycotoxin production and sporulation in several mycotoxigenic has been demonstrated: In *Fusarium verticillioides*, knocked out gene *FCC1* (for *Fusarium cyclin C1*) result in reduced production of conidia and fumonisin B_1_ biosynthesis (Shim and Woloshuk, 2001). Earlier studies suggest that deletion of *laeA* and *veA* genes in *A. alternata*, respectively, strongly reduced sporulation and greatly compromised mycotoxin production during pathogenesis of tomato fruits and *in vitro* (Estiarte et al., 2016). Consistent with these findings, biosynthesis of toxic secondary metabolites and sporulation in Δ*CcPtc1* showed reduced compared with WT strains in *C. chrysosperma*. Thus, the results suggest that terpene cyclase-like 2 protein *CcPtc1* plays an important role in the development of *C. chrysosperma*. Furthermore, although the effect of secondary metabolic genes on sporulation was not conserved, based on the previous articles and our results, we deduced that there was some connection between fungal toxin output and the sporulation process.

As mentioned earlier, necrotrophic pathogens tend to kill host cells rapidly. To date, several reports have demonstrated that secondary metabolite biosynthetic genes and the toxins they synthesize are the primary weapons of necrotrophic pathogens. For instance, deletion of *FgVEA* led to inhibition in aerial hyphae formation, virulence and deoxynivalenol (DON) biosynthesis in *F. graminearum* (Jiang et al., 2011). Another well-known toxin botrydial, produced by botrydial biosynthetic genes of *B. cinerea* could induce chlorosis and collapse of French bean tissue (Deighton et al., 2001; Siewers et al., 2005). Currently in *V. mali*, there have been many secondary metabolic genes that play a role in the infection process, such as *VmLaeA, VmVeA*, and *VmVelB* (Wu et al., 2018; Feng et al., 2020). In this study, we showed that secondary metabolite backbone gene *CcPtc1* markedly affected virulence and toxic secondary metabolites in the necrotrophic fungus *C. chrysosperma*. In addition, we showed that *C. chrysosperma* was likely to secrete a series of metabolites to damage the host tissue, and absence of *CcPtc1* would affect production of metabolites in *C. chrysosperma*. Further, combining previous studies with our findings, it is reasonable to speculate that toxins synthesized by secondary metabolic genes play an important role in the infestation process of necrotrophic pathogens.

In this study, untargeted metabolomics analyses revealed that pantothenate and CoA biosynthesis pathway was enriched. In pantothenate and CoA biosynthesis pathway, we identified two metabolites (pantothenate and pantothenol), and pantothenate was significantly down-regulated in Δ*CcPtc1* compared with the WT strain. Previous studies have showed that pantothenate (vitamin B5) is not only an essential metabolite for all biological systems, but also the precursor of the indispensable cofactor CoA (Alberts and Vagelos, 1966; Webb et al., 2004; Leonardi et al., 2005). Pantothenate plays dominant role in abundant biological processes, including carbohydrate, lipid, and nucleic acid metabolism. These reports were consistent with our metabolome results (Figure 6). Here, we found that the majority of DAMs were lipid, while a portion was carbohydrates. Therefore, we deduced that the level of pantothenate affects the process of toxin production in *C. chrysosperma* by influencing the metabolism of lipid and carbohydrates. In addition, we also found that pantothenate and pantothenate biosynthetic pathway shows critical impacts on virulence in *Histoplasma capsulatum* (Garfoot et al., 2014), *A. fumigatus* (Dietl et al., 2018) and *Mycobacterium tuberculosis* (MTB) (Sambandamurthy et al., 2002), and also necessary for the growth of bacterial (Srinivas et al., 2012; He et al., 2018; Yao et al., 2018). These results were identical to the defect of fungal development and pathogenicity in Δ*CcPtc1*, which suggest that *CcPtc1* possibly affected virulence and hyphae development by partial regulating the content of downstream metabolites in *C. chrysosperma*. Moreover, pantothenol (Dexpanthenol) has been reported to be capable of acting as a substrate for pantothenate kinase in MTB to produce 4′-phosphopantothenol and eventually affects the biosynthesis of CoA (Kumar et al., 2007). Our data indicated that the content of panthenol was significantly up-regulated in Δ*CcPtc1* compared with the WT strain. Therefore, we hypothesized that, *CcPtc1* might affects the biological reaction using CoA as cofactor by affecting the early intermediates of CoA synthesis, pantothenate and pantothenol (Figure 9B).

In the present study, we also found that another metabolite associated with fungal pathogenicity, trehalose 6-phosphate (T6P), accumulated significantly in the starch and sucrose metabolism pathway pathway. T6P is the key intermediate of the trehalose biosynthesis pathway in plants and yeast (Lunn et al., 2014; Figueroa and Lunn, 2016). Previous studies have determined that the accumulation of T6P has cytotoxic effects in different species (Puttikamonkul et al., 2010; Song et al., 2014; Korte et al., 2016). Additionally, T6P has been verified to have important impacts on the fungal virulence and development of filamentous fungal plant pathogens such as *Magnaporthe grisea* (Wilson et al., 2007), *M. oryzae* (Chen et al., 2021), and *Stagonospora nodorum* (Lowe et al., 2009). In the plant pathogenic fungus *M. grisea*, T6P synthase (Tps1) is a central regulator for regulating the pentose phosphate pathway and intracellular levels of NADPH, and responsible for fungal virulence (Wilson et al., 2007). Deletion of *MoTPS2* result in significantly intracellular accumulation of T6P, which leading to defects in the hyphae development and pathogenicity of the *M. oryzae* (Chen et al., 2021). Our data demonstrated that T6P was significantly accumulated in the Δ*CcPtc1* mutant compared with the WT strain. Correspondingly, the Δ*CcPtc1* mutant showed reduced pathogenicity and impaired growth. In *C. chrysosperma*, whether the accumulation of T6P is related to the defected pathogenicity and the affected hyphal growth of the Δ*CcPtc1* needs to be further verified.

As mentioned above, we also enriched some of organic acids and derivatives. Compared to the WT group, a total of 13 differentially abundant organic acids and derivatives metabolites were identified in Δ*CcPtc1* group, among which seven upregulated and six downregulated. Fungal pathogens are capable to be classified into two categories, acidic and alkaline fungi by whether fungal pathogens can acidify or alkalize the host’s environment by secreting organic acids or ammonia (Prusky and Lichter, 2008). Organic acids are essential in the invasion and pathogenesis of acidic fungi. Acidic fungi can acidify the host’s environment by secreting organic acids to enhancing their infectivity. Not only that, organic acid molecules secreted by acidic fungi plays a pivotal role in the activation of virulence factors and enhancement of pathogenicity of certain fungi (Jiao et al., 2022). Several studies have shown that organic acids such as citric, oxalic, and gluconic acids are participates in numerous pathogenic processes and important for fungal pathogenicity. Notably, supportive evidence of oxalic acid (OA) acting as a vital virulence factor in *C. chrysosperma* has been proved. Meanwhile it was substantialized that acidification of host environment was a necessary condition for pathogenic of *C. chrysosperma* (Wang and Wang, 2020). The metabolomic data provided evidence that *CcPtc1* played an influential role to some of the organic acids and derivatives. However, whether the effect of *CcPtc1* on the pathogenicity of *C. chrysosperma* was related to the downstream organic acids and derivatives needs to be further verified.

In summary, *CcPtc1* was one of the terpene cyclase-like 2 protein family members identified in *C. chrysosperma*, which was important for virulence, mycotoxin production and development.

## Data availability statement

The datasets presented in this study can be found in online repositories. The names of the repository/repositories and accession number(s) can be found in the article/Supplementary material.

## Author contributions

DX, CT, and YY designed the experiments. YY, LY, and XQ performed the experiments and the data analyses. YY prepared the figures and wrote the manuscript. All authors contributed to the article and approved the submitted version.

## Footnotes

1
http://gsds.cbi.pku.edu.cn/

2
http://www.ebi.ac.uk/interpro/

3
https://www.genome.jp/kegg/pathway.html

4
https://hmdb.ca/metabolites

5
http://www.lipidmaps.org/
