# Supplementary figures and images for: A putative terpene cyclase gene (CcPtc1) is required for fungal development and virulence in Cytospora chrysosperma
Source: Front Microbiol. 2023 Feb 20;14:1084828. doi: 10.3389/fmicb.2023.1084828 (PMC9986285; doi:10.3389/fmicb.2023.1084828)

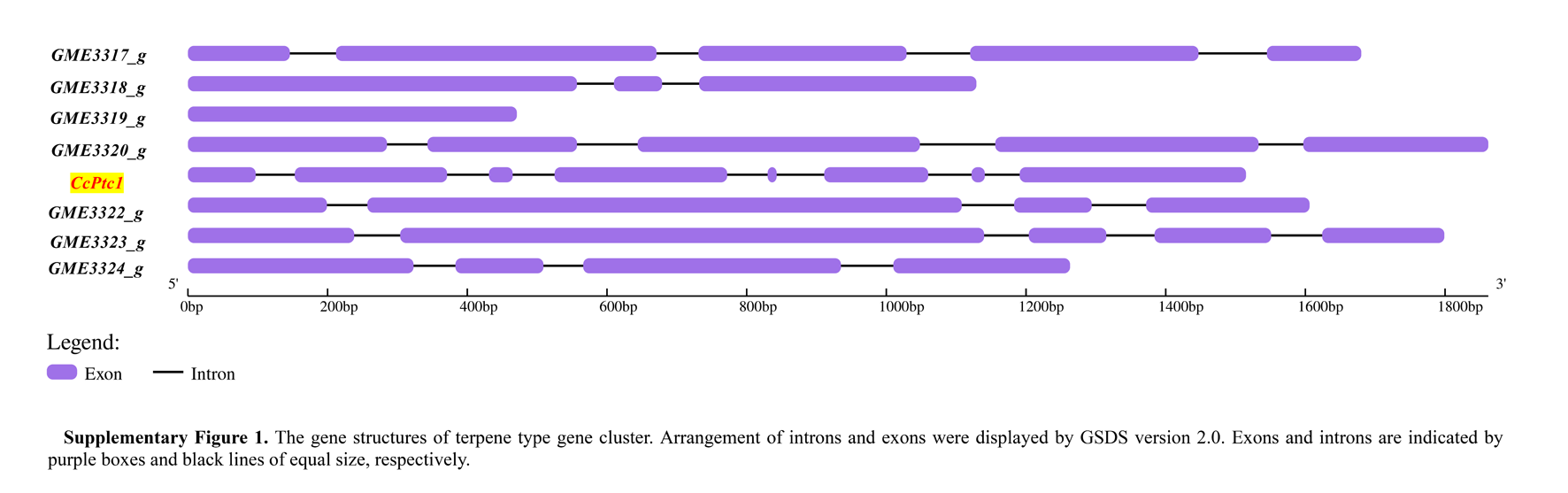

Supplement: Supplementary file 1 [file Image_1.TIF]

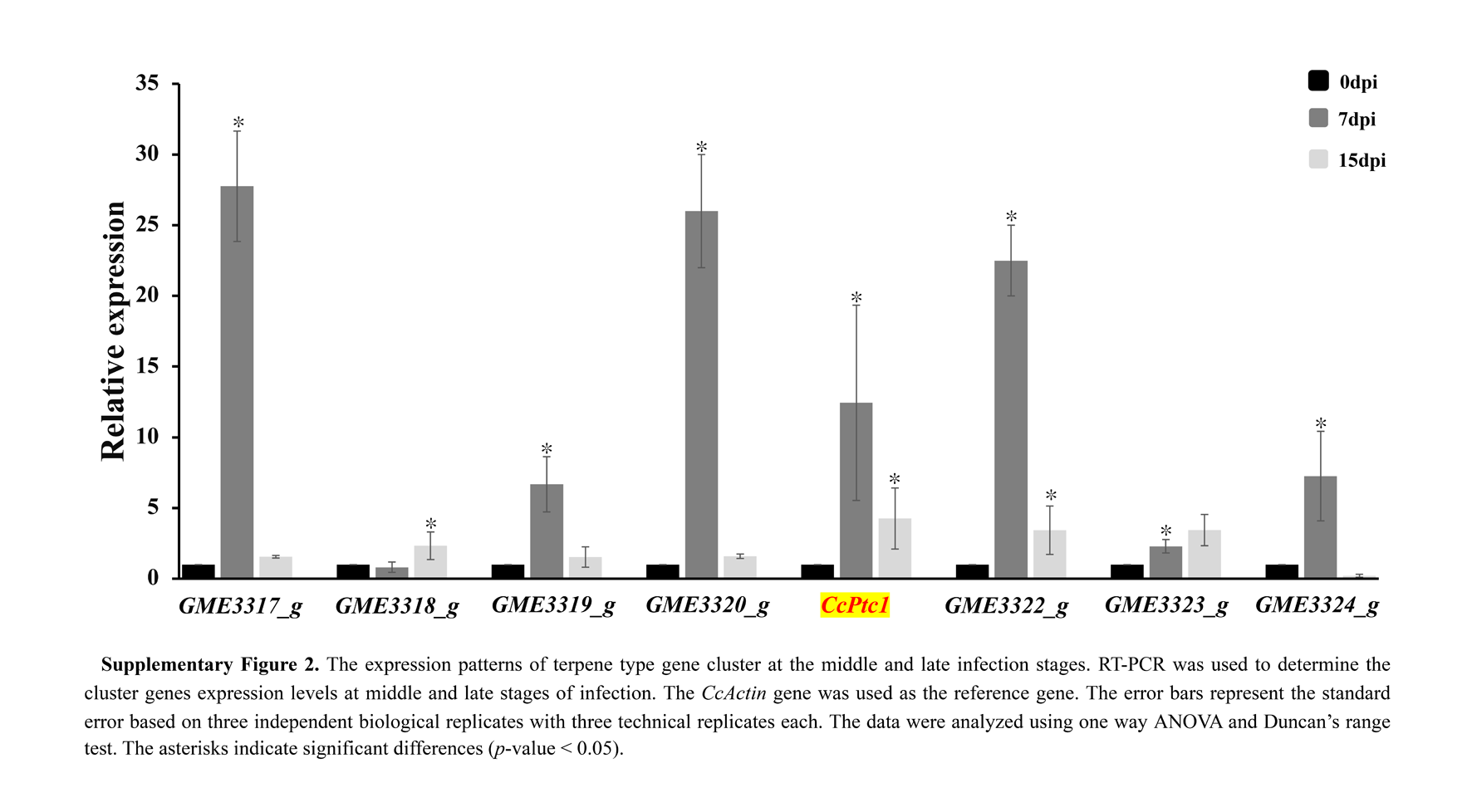

Supplement: Supplementary file 2 [file Image_2.TIF]

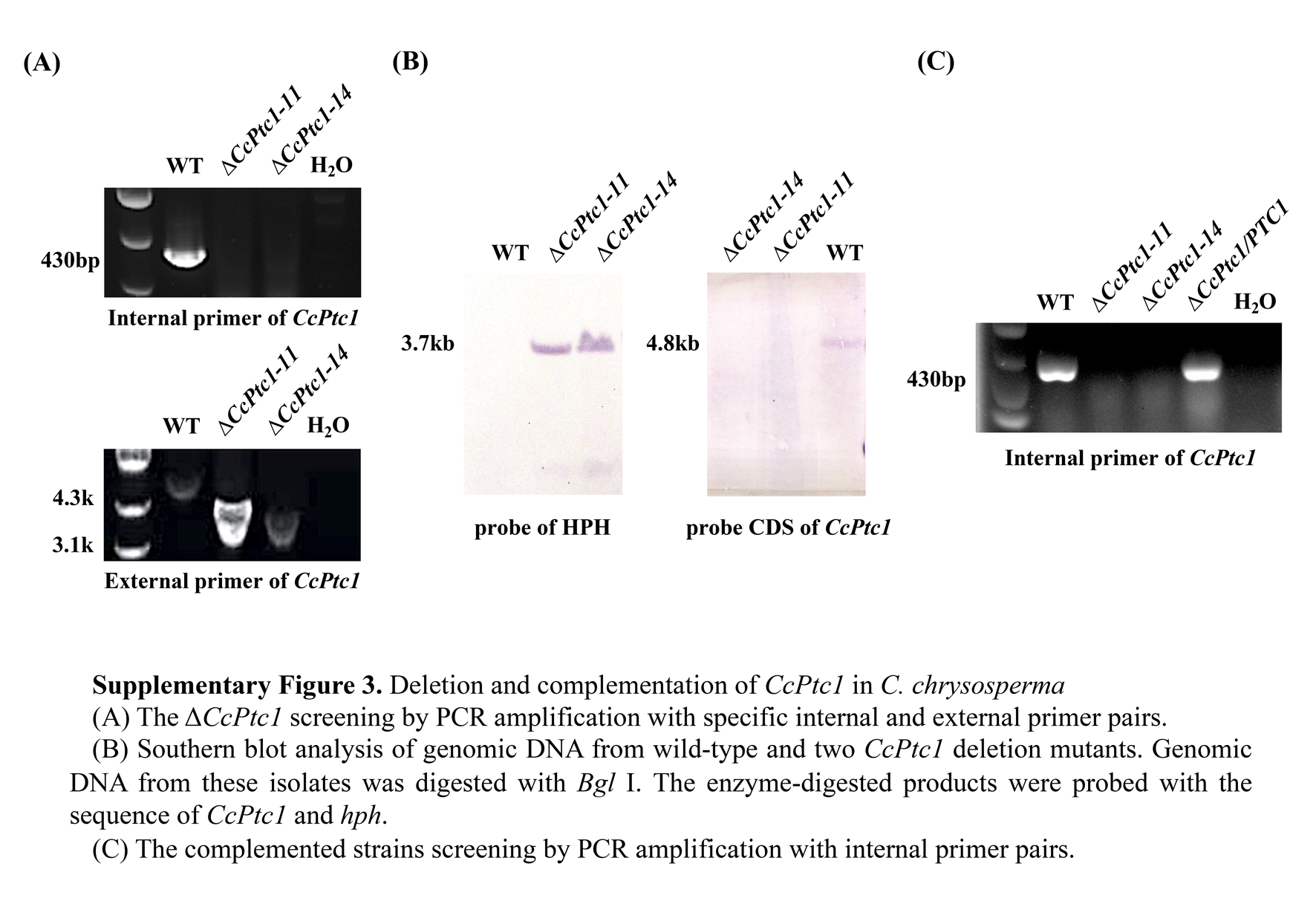

Supplement: Supplementary file 3 [file Image_3.TIF]
